# Epidemiology and Reporting Characteristics of Systematic Reviews

**DOI:** 10.1371/journal.pmed.0040078

**Published:** 2007-03-27

**Authors:** David Moher, Jennifer Tetzlaff, Andrea C Tricco, Margaret Sampson, Douglas G Altman

**Affiliations:** 1 Chalmers Research Group, Children's Hospital of Eastern Ontario Research Institute, Ottawa, Canada; 2 Department of Paediatrics, Faculty of Medicine, University of Ottawa, Ottawa, Canada; 3 Department of Epidemiology and Community Medicine, Faculty of Medicine, University of Ottawa, Ottawa, Canada; 4 Institute of Population Health, University of Ottawa, Ottawa, Canada; 5 Centre for Statistics in Medicine, Oxford, United Kingdom; UK Cochrane Center, United Kingdom

## Abstract

**Background:**

Systematic reviews (SRs) have become increasingly popular to a wide range of stakeholders. We set out to capture a representative cross-sectional sample of published SRs and examine them in terms of a broad range of epidemiological, descriptive, and reporting characteristics, including emerging aspects not previously examined.

**Methods and Findings:**

We searched Medline for SRs indexed during November 2004 and written in English. Citations were screened and those meeting our inclusion criteria were retained. Data were collected using a 51-item data collection form designed to assess the epidemiological and reporting details and the bias-related aspects of the reviews. The data were analyzed descriptively. In total 300 SRs were identified, suggesting a current annual publication rate of about 2,500, involving more than 33,700 separate studies including one-third of a million participants. The majority (272 [90.7%]) of SRs were reported in specialty journals. Most reviews (213 [71.0%]) were categorized as therapeutic, and included a median of 16 studies involving 1,112 participants. Funding sources were not reported in more than one-third (122 [40.7%]) of the reviews. Reviews typically searched a median of three electronic databases and two other sources, although only about two-thirds (208 [69.3%]) of them reported the years searched. Most (197/295 [66.8%]) reviews reported information about quality assessment, while few (68/294 [23.1%]) reported assessing for publication bias. A little over half (161/300 [53.7%]) of the SRs reported combining their results statistically, of which most (147/161 [91.3%]) assessed for consistency across studies. Few (53 [17.7%]) SRs reported being updates of previously completed reviews. No review had a registration number. Only half (150 [50.0%]) of the reviews used the term “systematic review” or “meta-analysis” in the title or abstract. There were large differences between Cochrane reviews and non-Cochrane reviews in the quality of reporting several characteristics.

**Conclusions:**

SRs are now produced in large numbers, and our data suggest that the quality of their reporting is inconsistent. This situation might be improved if more widely agreed upon evidence-based reporting guidelines were endorsed and adhered to by authors and journals. These results substantiate the view that readers should not accept SRs uncritically.

## Abstract 2

Data were collected on the epidemiological, descriptive, and reporting characteristics of recent systematic reviews. A descriptive analysis found inconsistencies in the quality of reporting.

## Editors' Summary

**Background.:**

In health care it is important to assess all the evidence available about what causes a disease or the best way to prevent, diagnose, or treat it. Decisions should not be made simply on the basis of—for example—the latest or biggest research study, but after a full consideration of the findings from all the research of good quality that has so far been conducted on the issue in question. This approach is known as “evidence-based medicine” (EBM). A report that is based on a search for studies addressing a clearly defined question, a quality assessment of the studies found, and a synthesis of the research findings, is known as a systematic review (SR). Conducting an SR is itself regarded as a research project and the methods involved can be quite complex. In particular, as with other forms of research, it is important to do everything possible to reduce bias. The leading role in developing the SR concept and the methods that should be used has been played by an international network called the Cochrane Collaboration (see “Additional Information” below), which was launched in 1992. However, SRs are now becoming commonplace. Many articles published in journals and elsewhere are described as being systematic reviews.

**Why Was This Study Done?:**

Since systematic reviews are claimed to be the best source of evidence, it is important that they should be well conducted and that bias should not have influenced the conclusions drawn in the review. Just because the authors of a paper that discusses evidence on a particular topic claim that they have done their review “systematically,” it does not guarantee that their methods have been sound and that their report is of good quality. However, if they have reported details of their methods, then it can help users of the review decide whether they are looking at a review with conclusions they can rely on. The authors of this *PLoS Medicine* article wanted to find out how many SRs are now being published, where they are being published, and what questions they are addressing. They also wanted to see how well the methods of SRs are being reported.

**What Did the Researchers Do and Find?:**

They picked one month and looked for all the SRs added to the main list of medical literature in that month. They found 300, on a range of topics and in a variety of medical journals. They estimate that about 20% of reviews appearing each year are published by the Cochrane Collaboration. They found many cases in which important aspects of the methods used were not reported. For example, about a third of the SRs did not report how (if at all) the quality of the studies found in the search had been assessed. An important assessment, which analyzes for “publication bias,” was reported as having been done in only about a quarter of the cases. Most of the reporting failures were in the “non-Cochrane” reviews.

**What Do These Findings Mean?:**

The authors concluded that the standards of reporting of SRs vary widely and that readers should, therefore, not accept the conclusions of SRs uncritically. To improve this situation, they urge that guidelines be drawn up regarding how SRs are reported. The writers of SRs and also the journals that publish them should follow these guidelines.

**Additional Information.:**

Please access these Web sites via the online version of this summary at http://dx.doi.org/10.1371/journal.pmed.0040078.

- An editorial discussing this research article and its relevance to medical publishing appears in the same issue of *PLoS Medicine*
- A good source of information on the evidence-based approach to medicine is the James Lind Library
- The Web site of the Cochrane Collaboration is a good source of information on systematic reviews. In particular there is a newcomers' guide and information for health care “consumers”. From this Web site, it is also possible to see summaries of the SRs published by the Cochrane Collaboration (readers in some countries can also view the complete SRs free of charge)
- Information on the practice of evidence-based medicine is available from the US Agency for Healthcare Research and Quality and the Canadian Agency for Drugs and Technologies in Health

## Introduction

Systematic reviews (SRs) have become increasingly popular in medicine. Clinicians read them as an efficient way of keeping up-to-date with their content area. Clinical practice guideline developers use them as a starting point for guideline development. Granting agencies require them as an evidence base for the need to conduct new research [1], and healthcare journals are moving in the same direction [2]. These different stakeholders should be encouraged to use SRs, particularly if they are of high quality and have taken steps to minimize bias.

The principal opportunity to estimate how a SR was designed and conducted is by examining its report. In one of the first evaluations of SRs, in 1987 Sacks and colleagues examined 83 SRs regarding their adequacy of reporting, using 23 characteristics covering six domains [3]. The authors noted that reporting was generally poor, with between 1 and 14 characteristics adequately reported (mean = 7.7; standard deviation = 2.7). For example, only six (7%) of the 83 reports mentioned use of a protocol to conduct the SR. A 1999 update [4] of a 1987 publication [5] found little improvement in the quality of reporting of SRs over time. Others have recently reported similar results, namely, that the quality of reporting of SRs is less than optimal [6,7].

Some possible limitations of these evaluations are that they did not set out to examine epidemiological aspects of SR reports, such as their publication prevalence and different types of SRs being published, nor did they examine a representative sample of published SRs. Typically the focus of such examinations is in specific health care areas [8–11] or type of SR [12,13]. Also, some of these evaluations are relatively old and thus could not address new and emerging issues relevant for SRs, such as the frequency with which they are updated. Our sampling strategy allowed us to comprehensively examine recent SRs across all application areas.

We set out to capture a cross-sectional sample of all recently published SRs and examine them in terms of a broad range of epidemiological and reporting characteristics, including emerging issues not previously examined.

## Methods

### Definition

There is no standard definition of an SR. We counted a report as an SR if the authors' stated objective was to summarize evidence from multiple studies and the article described explicit methods, regardless of the details provided.

### Search

We believed that one month's publications would provide a few hundred eligible studies that would be enough to give a reliable summary of the literature. The Cochrane library is reissued quarterly. November 2004 was selected as it was the most recent month including new Cochrane reviews.

MEDLINE was searched to identify potential systematic reviews entered into Medline in November 2004. We used Montori's balanced five-term search strategy (specificity > sensitivity [14]), and supplemented it with his balanced three-term strategy (sensitivity > specificity), modified slightly: Ovid MEDLINE 1966 to Feb Week 1 2005 (searched Feb 18 2004), (1) 200411$.ed; (2) limit 1 to English (3) 2 and (cochrane database of systematic reviews.jn. or search.tw. or meta-analysis.pt. or medline.tw. or systematic review.tw. or ((meta-analysis.mp,pt. or review.pt. or search$.tw.) and methods.ab.))

In the case of the three-term strategy we required that the string “methods” appear in the abstract. We further limited the search to SRs reported in English due to the additional resources required to translate SRs published in other languages. The search results were uploaded into an Internet-based management program (Systematic Review System).

### Screening

Two members of the research team independently screened the citations (title and abstract). One reviewer subsequently screened the full text articles of citations meeting our definition and those in which eligibility remained unclear. A second reviewer independently screened a 10% random sample. When it was clear that the intent of the authors was a literature review (e.g., authors identified the review as a brief overview with no specific review question), as opposed to an SR, articles were excluded. All other articles were included.

### Data Collection and Analysis

Data collection was completed using a form that included 51 questions. Here we summarize a subset focusing on epidemiological and descriptive characteristics, including those with a potential for bias (Tables 1 and 2). We classified the Cochrane Database of Systematic Reviews, where Cochrane reviews are published, as a specialty journal. Reviews were classified according to their focus: therapeutic (e.g., treatment or prevention), diagnostic/prognostic (e.g., clinical prediction rules), epidemiology (e.g., incidence, prevalence), or other (e.g., education).

**Table 1:**
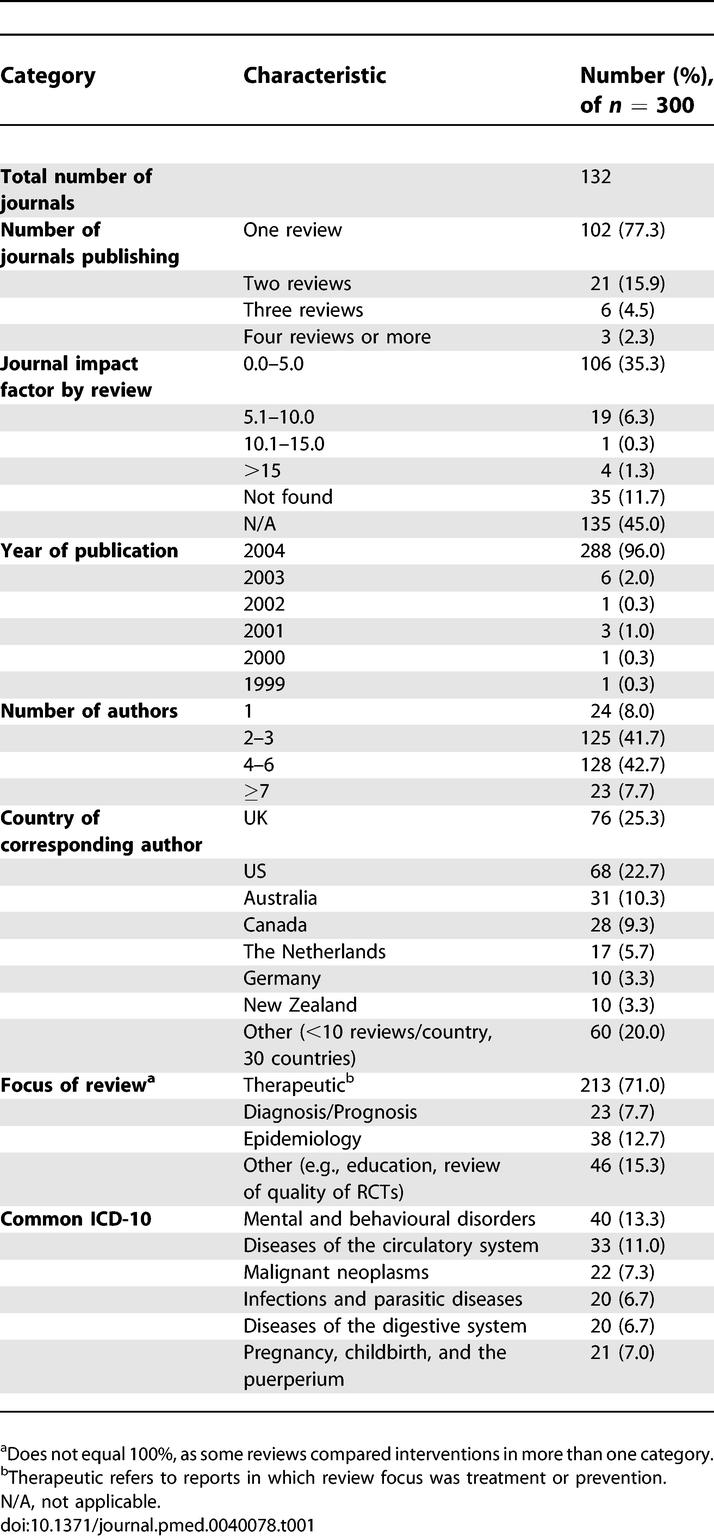
Epidemiology of Systematic Reviews

**Table 2:**
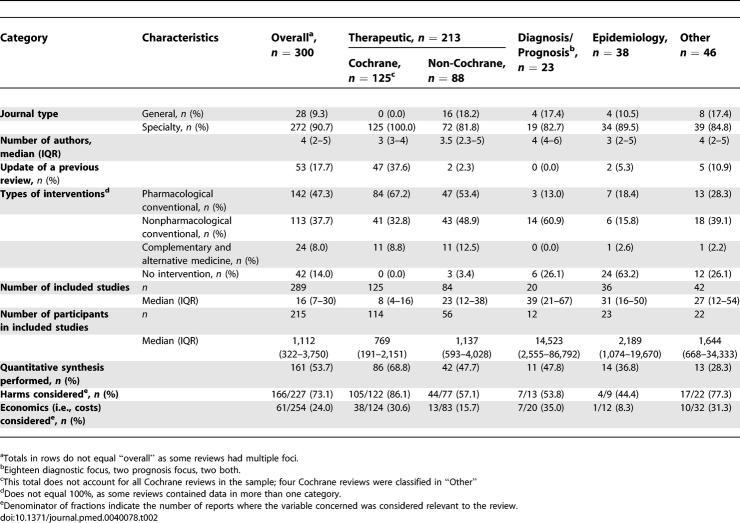
Descriptive Characteristics of Included Systematic Reviews

Two reviewers independently pilot-tested the form on a separate set of five articles. Each article was screened independently by one of three team members with a 5% sample screened in duplicate by two reviewers. Any uncertainties were discussed among the data extractors, and conflicts were resolved by coming to consensus.

All data analyses were performed using SPSS (version 13; http://www.spss.com). The analysis was descriptive. Data are summarized as frequency or median and interquartile range (IQR). We completed the one a priori subgroup analysis comparing Cochrane reviews to non-Cochrane reviews in terms of their reporting characteristics with a potential for bias.

## Results

### Search

**Figure 1:**
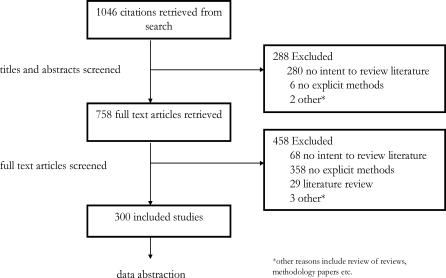
Flow of Citations through the Cross-Sectional Identification and Retrieval of Systematic Reviews Indexed in Medline during November 2004

Our search identified 1,046 records (Figure 1). Initial screening excluded 291 records, including two duplicates. The remaining 758 full-text articles were retrieved for additional scrutiny, of which 458 proved ineligible. Agreement was excellent between reviewers across the screening phases (kappa = 0.86). The majority of articles (78.2%) were excluded because they did not report any methods explicitly. We thus included 300 eligible reports for full data extraction.

### Prevalence of Publishing SRs

We identified 300 SRs that were indexed in Medline in November 2004. Cochrane reviews contributed nearly half of these reviews (125 [41.7%]). Given that Cochrane reviews are published and indexed quarterly, this corresponds to an estimated annual frequency of approximately 2,500 publications, of which about 20% are Cochrane reviews. Although the SRs were all indexed in November 2004 they had been published between 1999 and 2004 (2004 = 96%). One review was published in the Cochrane Library and a paper-based journal; both are included here.

Using our definition of an SR we had difficulty in deciding on whether 30 (10%) of the included reviews were actually systematic. We completed a post-hoc sensitivity analysis with and without these 30 reviews. The results did not change in a meaningful way. As such all of the analyses presented here include all 300 SRs.

### Overall Epidemiology Characteristics

The 300 reviews were published in 132 journals, with most journals publishing only one SR indexed during the month although three journals each published four or more SRs (Table 1). Most reviews were published in journals with impact factors of five or less, although four SRs were published in journals with a high impact factor (>15). Nearly half of the SRs (135 [45%]), including all of the Cochrane SRs, did not have an impact factor. The reviews included a median of four authors although 24 (8%) of the reviews were single authored (Tables 1 and 2). One-quarter (76 [25.3%]) of the corresponding authors were from the United Kingdom with four countries (UK, US, Australia, and Canada) accounting for two-thirds of the SRs published during the month (Table 1). The majority of the reviews (213/300 [77%]) were classified as therapeutic—of which slightly more than half (125/213 [59%]) were Cochrane reviews—38/300 (13%) as epidemiology reviews, and 23/300 (8%) as diagnostic/prognostic reviews. Forty-six (15%) of the 300 reports were categorized as other, such as assessing the quality of immunosuppression trials in kidney transplantation [15].

Nearly half (142 [47.3%]) of the reviews reported examining the health effects of pharmacological interventions with the only a small percentage (24 [8.0%]) of reviews reported examining complementary and alternative medicine. The most common review classification (International Classification of Diseases 10 [ICD-10]) was mental and behavioural disorders (40 [13.3%]).

### Descriptive Characteristics

Almost all of the SRs (272 [90.7%]) were published in specialty journals (Table 2). Few reviews (53 [17.7%]) reported being updates of previously completed reviews, although, of the therapeutic reviews, more than one-third of the Cochrane ones were updates (47/125 [37.6%]); almost no non-Cochrane reviews were reported as updates (2 [2.3%]). The SRs included a median of 16 studies involving 1,112 participants, although this number varied considerably by review category. Therapeutic reviews were the smallest and, of these, Cochrane reviews included fewer studies (median = 8) and participants (median = 769) compared to non-Cochrane reviews (median number of studies = 23; median number of participants = 1,137). Those reviews with a diagnostic/prognostic focus were the largest by far (Table 2). One hundred sixty-one reviews (53.7%) reported combining the results of their included studies statistically (i.e., meta-analytical synthesis). Most reviews (166/227 [73.1%]) reported on some aspect of harms. However, few reviews (61/254 [24%]) considered economic issues in their report.

### Reporting Characteristics Related to Potential for Bias

Half (150 [50%]) of the review reports used the term “systematic review” or “meta-analysis” in the title or abstract with little variation across review category. However, of the therapeutic reviews, Cochrane reviews were less likely to use “systematic review” and/or “meta-analysis” in the title or abstract (49 [39.2%]) compared to non-Cochrane reviews (60 [68.2]%). Although close to half (139 [46.3%]) of the reviews reported working from a protocol, therapeutic reviews were more likely to report using a protocol than were other types of reviews (Table 3). Within the therapeutic review category almost every Cochrane review reported a protocol (122/125 [97.6%]), but only a small minority of non-Cochrane reviews did (10/88 [11.4%]).

**Table 3:**
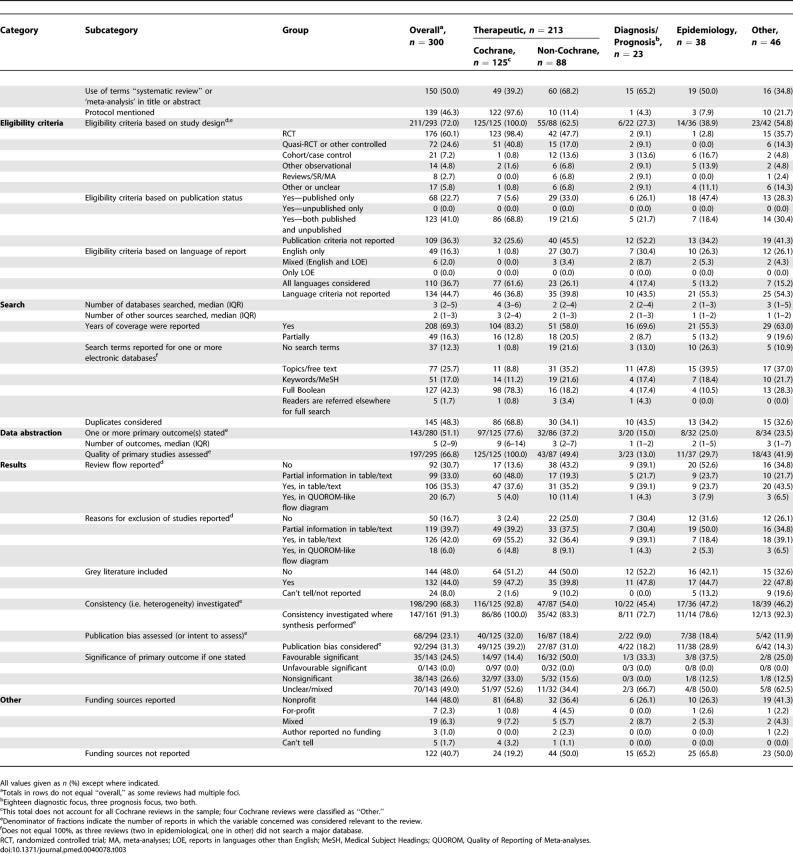
Descriptive and Reporting Characteristics with Potential for Bias

The majority (211/293 [72.0%]) of reviews reported study design eligibility criteria, although this varied across and within review category. Of the therapeutic reviews almost all of Cochrane ones included randomized controlled trials (RCTs) only (123/125 [98.4%]), whereas slightly less than half of the non-Cochrane reviews had such restrictions (42/88 [47.7%]). Between 45% and 73% of the reviews in other categories did not specify any study design eligibility criteria (Table 3). About one-third of the reviews (109 [36.3%]) did not report whether they had any publication status eligibility criteria. Those reviews that did provide this information tended to include both published and unpublished documents (123 [41%]). Nearly half (134 [44.7%]) of the SRs did not report whether they had eligibility criteria based on language of reports. Of those that did provide this information, most (110 [36.7%]) included all languages (Table 3). Of the therapeutic reviews, only one Cochrane review (1/125 [0.8%]) reported limiting to reports of studies written in English, whereas about one third of non-Cochrane reviews reported this information (27/88 [30.7%]).

Reviews reported searching a median of three electronic databases and two other sources, although therapeutic reviews reported searching more sources than did other types of reviews (Table 3). For therapeutic reviews, Cochrane SRs typically searched twice as many databases compared to non-Cochrane SRs (median: four versus two, respectively). Approximately two-thirds (208 [69.3%]) of the reviews reported on the years covered for their electronic searches. There was little consistency concerning the electronic search information provided in SR reports. Of the therapeutic reviews, the majority of Cochrane reviews (98/125 [78.3%]) provided a full Boolean strategy, whereas only a minority of non-Cochrane reviews (16/88 [18.2%]) provided this information. Search strategies were reported in 10.9%–26.3% for the other review categories (Table 3). About half (145 [48.3%]) of the reviews reported on whether they considered duplicates as part of their overall approach to searching. For therapeutic reviews, twice as many Cochrane SRs (86/125 [68.8%]) considered duplicates compared to non-Cochrane reviews (30/88 [34.1%]).

Approximately half (143/280 [51.1%]) of the reviews reported at least one primary outcome, with reviews typically reporting a median of five outcomes, although therapeutic reviews reported nearly double this number (median = 9; see Table 3). For therapeutic reviews, the majority of Cochrane ones reported a primary outcome (97/125 [77.6%]), of which only a minority (14/97 [14%]) reported a statistically favourable result for this outcome whereas only about a third of non-Cochrane reviews reported a primary outcome (32/86 [37.2%]), of which half reported a statistically favourable result (16/32 [50%]). Two-thirds of the reviews (197/295 [66.8%]) reported information about quality assessment of their studies. For therapeutic reviews, all the Cochrane ones reported assessing the quality of included studies whereas only half of the non-Cochrane did (43/87 [49.4%]).

Most reviews (208 [69.3%]) reported on the flow of information throughout the review process, although there was little consistency as to how this flow was reported, with only a minority (20 [6.7%]) including the use of a QUOROM-like flow diagram (Table 3). Most reviews (250 [83.3%]) reported some information concerning the reasons for excluding studies, although, like the flow of information through the review, there was little consistency and completeness in how exclusions were reported (Table 3). Grey literature was reported to be included in 132 (44%) of SRs with minimal differences across the review categories or within the therapeutic review category (Table 3).

Of those reviews reporting a quantitative synthesis, the vast majority (147/161 [91.3%]) reported assessing for consistency across the studies. However, less than one-quarter of the reviews (68/294 [23.1%]) reported assessing for publication bias. For therapeutic reviews, about twice as many Cochrane SRs (40/125 [32.0%]) reported assessing for publication bias compared to non-Cochrane reviews (16/87 [18.4%]). Funding sources were not reported in 122 (40.7%) of the reviews. A small number of reviews (7 [2.3%]) reported being funded by for-profit sources (Table 3). Of the therapeutic reviews that reported funding source information, nearly twice as many were Cochrane reviews (101/125 [80.8%]) compared to non-Cochrane reviews (44/88 [50%]). No SR in our sample reported a registration number.

## Discussion

Our study shows that SRs are now being produced in large numbers, with about 2,500 new publications indexed annually on Medline, of which about one-fifth are Cochrane reviews. This value is an underestimate of the total number of new non-Cochrane SRs, as we examined only a single database and our searching might not have identified all SRs, and we restricted our study to English-language publications.

Our results indicate that SRs predominantly address questions about the effectiveness of interventions, and about half of them report combining their results statistically. Recent SRs include more studies and participants than previously reported [4–6], and while Cochrane reviews are smaller than other therapeutic reviews in our sample, their size corresponds to more recent data on Cochrane reviews [16]. Similarly, and like randomized trials [17], SRs are typically published in specialty journals.

There are some areas in which the quality of reporting has improved over time, such as the use of protocols [3] and quality assessment [18]. Similarly, very few reviews reported being funded by commercial sources, for which such funding has been associated with bias in the results of clinical trials [19].

It is too early to comment on the significance of some of our findings. Few reviews are reported as updates of previously completed reviews. The utility of reviews likely diminishes over time as they become outdated. Likewise, no review had a formal registration number. Our study also provided some disappointing results. For example, publication bias was considered or assessed in only a minority of reports despite much evidence for its existence and potential influence on the results of reviews [20,21].

However, for therapeutic reviews our comparison of Cochrane and non-Cochrane reviews provides the most discouraging results and suggests little improvement in the quality of reporting of non-Cochrane reviews over time [4,7]. The Cochrane Collaboration has a strict set of policies and guidance as to how SRs should be conducted and reported. For example, it is Cochrane policy not to include the words “systematic review” or “meta-analysis” in the review title. This policy explains why we observed fewer Cochrane reviews reporting these terms, compared to non-Cochrane ones. Similarly, Cochrane reviews, because they are reported in an electronic medium, are not encumbered by word length restrictions as is the case for most paper-based journals. Electronic publication, along with mechanisms to ensure adherence to Cochrane policies, allows authors to provide more complete details of how they conducted their SRs, possibly explaining why we observed far superior reporting standards of Cochrane reviews compared to non-Cochrane therapeutic ones. Journal editors and/or readers, particularly of specialty journals, might be less interested in these details and/or not have the resources to monitor adherence to any set of policies and reporting guidelines.

Many non-Cochrane reviews did not report key aspects of SR methodology, thus impairing confidence in their results and conclusions. For example, only 11% of the authors reported working from a protocol to complete their review, showing little improvement over time [3]. An examination of 47 Cochrane reviews, for which protocols almost always exist, revealed that 43 (91.5%) of them reported a major change such as the addition or deletion of outcomes between the protocol and the full publication [22].

Strong evidence of outcome reporting bias was recently reported within clinical trials [23,24]. Our results suggest that some aspect of selective outcome reporting bias might also exist within non-Cochrane reviews. Only about one-quarter of them reported a primary outcome, of which half report statistical significance in favour of this outcome (versus 14.4% for Cochrane reviews). This issue requires further investigation.

Results from SRs are most useful when they are up-to-date [25]. Few reviews were reported as updates and there were large differences between Cochrane reviews and non-Cochrane reviews. This difference might reflect differing or nonexistent policies and/or practices across funders, but also may reflect the reluctance of journals to publish updates that are substantially the same as previous publications. If these SRs are to retain their currency, updating them needs to be a much higher priority. This issue is likely to become increasingly important in coming years.

Clinical trial registration was called for twenty years ago [26]. In an attempt to minimize or avoid recent questionable behaviours such as hiding data [27], it is now becoming widely endorsed by granting agencies, such as the Canadian Institutes of Health Research [1], and editorial groups, such as the International Committee of Medical Journal Editors [28]. Similar behaviours may well affect SRs, although there are currently few data to inform this belief. Although protocols of Cochrane reviews are published, no review in our sample was registered in the usual sense, or had any type of registration number. Systematic review registration options exist [29,30] but are not well known. However, the reasons for registering SRs are likely different from that of registering clinical trials.

Providing reporting guidance is one way to improve the above-mentioned deficiencies. Real improvements have been seen in the reporting of RCTs [31] since the introduction of the CONSORT Statement and endorsement by journals [32,33] and editorial groups (for example, http://www.icmje.org). The QUOROM Statement, which provides reporting guidance for meta-analysis of RCTs, was developed ten years ago in the hope of having a similar impact on meta-analysis [34], and there are some indications that its use is associated with improved quality of reporting [35]. Its uptake was much slower than was the CONSORT statement, possibly reflecting the belief that SRs were less frequently reported and not as important as RCTs. However, our study shows that currently about 2,500 such studies are published annually in English and indexed in Medline. QUOROM is currently being revised, and when published will provide journals and others with another opportunity to endorse its use as one way to improve the reporting of SRs. It is possible that the poor reporting of some SR categories reflects inadequate guidance available to authors. For example, we are unaware of any reporting guidance for SRs of prognostic studies.

Efforts directed toward improving reporting standards come at the end of the research process. Attempts to exert an influence much earlier in the research cycle might have a more profound and lasting effect. One option is to ensure that the evaluation process by granting agencies requires prospective applicants and grant peer reviewers to have some form of experience and expertise with SRs, for example by providing documentation of SR training. Additionally, journals might consider asking authors to include their SR protocols along with the completed review submissions.

Our cross-sectional analysis was limited in that we included just one month from a single database. It is unlikely, however, that sampling other months would have changed our results, as we verified that the month we chose was a typical entry month. While we included 300 reviews in our sample the majority of them were categorized as therapeutic. As such, our results concerning the other categories should be interpreted cautiously.

Our examination relied on what the authors reported. It is possible that the authors more completely conducted their SRs but omitted important details from their report, or the peer-review process resulted in the removal of key information we sought.

We have not provided here detailed information on several characteristics, such as the methods used to perform quantitative data synthesis or assess publication bias. More detailed information will be forthcoming in additional reports. Our intent here is to provide the reader with a broad overview of the reporting characteristics in recently published SRs.

Finally, we used a broad definition of what constitutes a SR. It is possible that a small proportion of our sample were intended to be literature reviews with very thorough descriptions of methods employed for the literature search. However, as such reviews would likely be used to the same extent as reports that are clearly defined as SRs or whose authors have quantitatively synthesized the information, we feel that they are subject to the same susceptibility to bias. Despite publishing SRs for about half a century, the National Library of Medicine does not yet index them as a publication type, at least in part because there is no agreed-upon definition of what constitutes a SR [36], although there are initiatives to help identify them [37]. A publication type for SRs on Medline is urgently needed.

## Abbreviations

IQR: interquartile range
RCT: randomized controlled trial
